# Improving cardiometabolic health assessments and interventions at St Charles Hospital, London

**DOI:** 10.1192/bjo.2021.571

**Published:** 2021-06-18

**Authors:** Mehtab Rahman, Vernanda Julien

**Affiliations:** Central & North West London NHS Foundation Trust

## Abstract

**Aims:**

St Charles is one of the largest inpatient mental health units in London with 8 wards and covers the boroughs of Kensington & Chelsea and Westminster. This project aimed was set up so that 95% of patients in St Charles Mental Health Centre would have a complete cardiometabolic health assessment by December 2020. This would include Weight, Smoking, Alcohol, Substance Use, Hypertension, Cholesterol and Diabetes assessments with necessary interventions recorded. The outcome of the intervention would improve overall physical health and life expectancy.

**Method:**

People with serious mental illness experience significantly worse physical health and shorter life expectancy of up to 10 to 15 years than the general population. CNWL is making Physical Health of patients in Mental Health Services a priority. Performance in this area has been challenging across the Trust because of:

- Buy in from clinicians.
- Staff did not feel empowered to discuss interventions with patients.
- High sickness and absence as a result of COVID was found to directly correlate with reduced physical health monitoring/recording.
- Lack of training in completing the SystmOne physical health template

The following cardiometabolic risk monitoring interventions were recorded on SystemOne (electronic documentation platform) and performance reviewed using Tableau : Weight, Smoking, Alcohol, Substance Use, Hypertension, Cholesterol and Diabetes assessments with necessary interventions recorded.

**Result:**

Prior to the commencement of this project, the wards in St Charles Mental Health Centre completed physical health assessments on roughly 8% of the patients in February 2020. The QI project was implemented in June 2020. By September 2020, physical health recording across 8 wards across St Charles had increased to 89% following successful implementation of the interventions.

**Conclusion:**

The following interventions resulted in a significant improvement in physical health cardiometabolic risk monitoring at a busy inpatient mental health setting:

- Monthly physical heath meetings to enable shared learning with ward doctors, nurses and healthcare assistants.
- Ongoing one-to-one and group support to train staff with completing and recording physical health assessments.
- Tableau Physical Health Report regularly reviewed with MDT during ward round meetings.
- Physical health leads given supernumerary days to run physical health clinics on the wards.
- Fortnightly Physical health monitoring meetings with the Director of Nursing and Head of Governance.

